# The addition of β-Hydroxy β-Methylbutyrate (HMB) to creatine monohydrate supplementation does not improve anthropometric and performance maintenance across a collegiate rugby season

**DOI:** 10.1186/s12970-020-00359-4

**Published:** 2020-05-27

**Authors:** Gerald T. Mangine, Trisha A. VanDusseldorp, Garrett M. Hester, Jennifer M. Julian, Yuri Feito

**Affiliations:** Exercise Science and Sport Management, Kennesaw State University, 520 Parliament Garden Way NW, 30144 Kennesaw, GA Georgia

**Keywords:** Sprinting kinetics, Strength, Creatine kinase, Cortisol, Athletes

## Abstract

**Background:**

Muscular damage sustained while playing rugby may hinder performance across a season. β-Hydroxy β-Methylbutyrate (HMB) may help attenuate muscle damage and maintain lean mass and performance. This study sought to determine the effect of combining HMB with creatine monohydrate supplementation on measures of stress and muscle damage, body composition, strength and sprinting kinetics throughout a rugby season.

**Methods:**

This double-blind, cross-over investigation recruited 16 male collegiate rugby players to provide resting blood samples and complete assessments of body composition, strength and sprinting performance prior to their fall season (PRE_FALL_). After testing, the athletes were matched for fat-free mass and assigned to consume one of two supplementation regimens for 6 weeks: 5 g HMB + 5 g creatine per day (HMB-Cr: 20.9 ± 1.1 years; 177 ± 2 cm; 88.4 ± 4.9 kg) or 5 g creatine + 5 g placebo per day (Cr: 21.4 ± 2.1 years; 179 ± 2 cm; 88.3 ± 4.9 kg). After 6 weeks (POST_FALL_), PRE_FALL_ testing was repeated in 13 of the original 16 athletes before a 10-wk wash-out period. Athletes who returned for the spring season (*n* = 8) repeated all fall-season procedures and testing prior to (PRE_SPRING_) and following (POST_SPRING_) their 6-wk spring season, except they were assigned to the opposite supplementation regimen.

**Results:**

Linear mixed models with repeated measures revealed group x time interactions (*p* <  0.05) for observed for several measures but did not consistently and positively favor one group. During the fall season, knee extensor peak torque was reduced by 40.7 ± 28.1 Nm (*p* = 0.035) for HMB-Cr but remained consistent for Cr, and no group differences or changes were noted in the spring. In the spring, greater knee flexor rate of torque development (~ 149 Nm·sec^− 1^, *p* = 0.003) and impulse (~ 4.5 Nm·sec, *p* = 0.022) were observed in Cr at PRE_SPRING_ but not at POST_SPRING_. Although significant interactions were found for cortisol concentrations, vastus lateralis pennation angle, and sprinting force, post-hoc analysis only revealed differences between fall and spring seasons. No other differences were observed.

**Conclusions:**

The combination of HMB and creatine monohydrate supplementation does not provide a greater ergogenic benefit compared to creatine monohydrate supplementation alone. Body composition, strength, and sprinting ability did not change across the season with creatine monohydrate supplementation.

## Background

At the Division 1-AA level, collegiate rugby players participate in matches and full-contact practices on a weekly basis across fall and spring seasons. Although being strong, fast, and powerful better equips a player to excel in their duties [1, 2], the duration and physical demands of a match and season may negatively impact these abilities. Athletes have been reported to experience serious structural damage and elevated markers of stress after a match and across a season [3–5], with the number of collisions [3] and intensity of training [4] being the most influential factors. Prolonged exposure to these conditions may lead to decrements in lean mass [6–9], which could also impact strength, speed, and power. Indeed, rugby athletes have reported greater overall feelings of fatigue and reduced physical performance after a season [5]. Nevertheless, players who maintain (or improve) their skills over the course of the season are more capable of contributing to their team’s late season success.

The availability of phosphocreatine (PCr) heavily influences strength, speed, and power [10]. As the duration or frequency of high-intensity activity (e.g., tackling, sprinting) increase, the PCr supply is depleted and performance is impaired due to an increased reliance on slower metabolic pathways [11–14]. Rugby athletes might delay this shift during a match, enhance recovery between matches, and improve performance by increasing intramuscular PCr via supplementation. Daily consumption of 3–5 g of creatine monohydrate has been extensively shown to improve a variety of athletic performance measures, including maximal strength and speed, as well as their recovery [10]. Supplementation may also indirectly reduce protein breakdown and muscle damage [15] and increase protein synthesis [16, 17] through muscular cell hyper-hydration, which would ultimately impact lean mass. Thus, creatine monohydrate remains as one of the most widely-used ergogenic supplements among athletes [10].

Nutritional supplementation with β-Hydroxy β-Methylbutyrate (HMB) may be another strategy for limiting muscle damage sustained during the season and maintaining athletic performance. As a metabolite of leucine, HMB is thought to positively impact protein balance [18–20] and thus, could impact lean mass and performance. A reduced creatine kinase, a biomarker for muscular damage, response was seen in men and women who participated in a progressive resistance training regimen and supplemented with 3 g of HMB for 4 weeks [21]. In trained athletes, 3 g of HMB supplementation for 4–12 weeks has been linked to improvements in athletic performance [22, 23] and lean mass [23, 24] in some, but not all studies [25–29]. It has been speculated that the degree of damage and stress associated with training influences the occurrence of a positive effect [30], for example, during periods of caloric restriction [31, 32]. The rigors of a rugby season may also satisfy this prerequisite, and combining HMB with creatine monohydrate, may elicit a more pronounced effect on performance due to their different proposed mechanisms of action [10, 18].

To the best of our knowledge, only a pair of studies have examined this hypothesis [33, 34] and both did not report performance advantages from the combination of HMB (3 g) and creatine monohydrate (3 g) compared to HMB alone or placebo. However, it is difficult to arrive at definitive conclusions from these data because the HMB dosage was at the low-end of the commonly investigated range (3–6 g) [35], recommended creatine loading procedures were not employed [10], the lack of participant blinding introduced bias, and the amount of demand from training was not quantified. Therefore, the purpose of this study was to study the combined effect of HMB and creatine supplementation compared to creatine supplementation alone on measures of biological stress and damage, body composition, strength, and sprinting ability across a collegiate rugby season. We hypothesized that over the course of the season, markers of biological stress and damage would be elevated, muscle size would decrease, and strength and sprinting performance would be impaired. However, the addition of HMB to creatine supplementation would attenuate these changes to a greater degree than creatine supplementation alone.

## Methods

### Experimental design

**Fig. 1:**
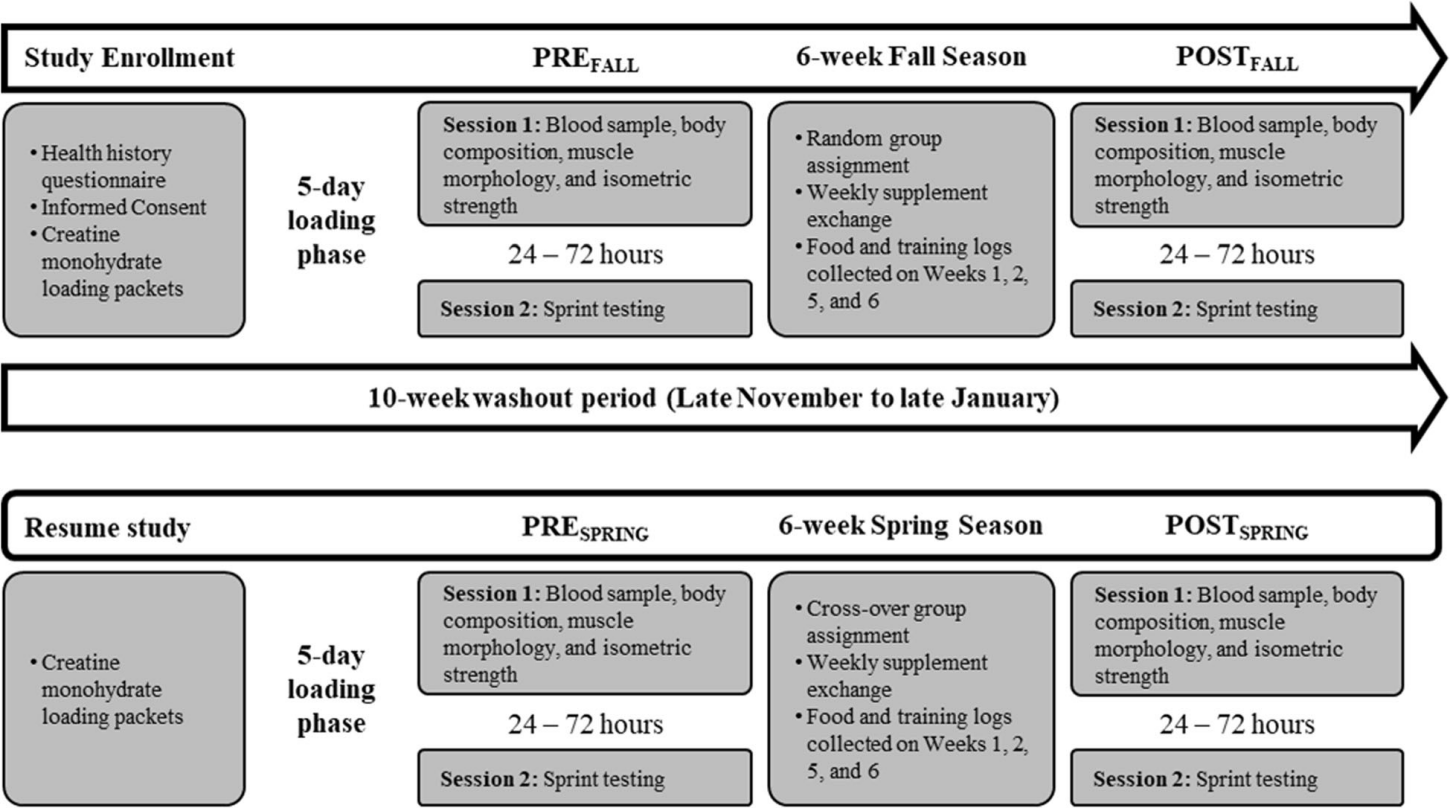
Study design and timeline

Collegiate, male rugby players were recruited for this double-blind, randomized investigation. Following a 5-day creatine monohydrate loading phase, all athletes arrived at the human performance laboratory (HPL) in the morning, fasted (8–10 h), and after having refrained from unaccustomed (i.e., extreme deviation from normal training) or vigorous exercise (48 h). The athletes initially provided a blood sample before completing assessments of body composition, muscle morphology, and isometric strength testing. Within 48–72 h, participants reported to their normal training facility for sprint testing, which completed all testing prior to the fall season (PRE_FALL_). The athletes were then matched for fat-free lean mass and randomly assigned to consume one of two supplementation regimens: HMB and creatine monohydrate (HMB-Cr) or creatine monohydrate and placebo (Cr) for 6 weeks. All PRE_FALL_-assessments were repeated following the 6-wk fall competitive season (POST_FALL_) and prior to- (PRE_SPRING_) and following (POST_SPRING_) the spring competitive season. Between the fall and spring seasons, the athletes completed a 10-wk wash-out period where they discontinued all supplementation. Prior to PRE_SPRING_, returning athletes once again completed a 5-day creatine monohydrate loading period before being reassigned to consume the opposite supplementation regimen from their fall assignment (i.e., cross-over design). All warm-ups and maximal efforts during strength and sprint testing were completed under the supervision of a Certified Strength and Conditioning Specialist. Figure 1 illustrates the study design and timeline.

### Participants

A priori analysis (effect size *f* = 0.25, ß = 0.80, α = 0.05) using sprinting performance as the most relevant and sport-specific measure indicated 16 participants would be necessary to observe an effect in this repeated-measures design. Following an explanation of all study procedures risks, and benefits, a convenience sample of 16 collegiate (Division 1-AA) American rugby players (21.1 ± 1.6 years [range = 19.0–24.2 years]; 178 ± 6 cm; 88.3 ± 14.2 kg) from the University’s team and who were free of any physical limitations (determined by medical history questionnaire and physical activity readiness questionnaire) provided their written informed consent to participate in this investigation. During the fall season, three athletes left the study prior to POST_FALL_ testing for the following reasons: personal, scheduling conflict, and illness (athlete self-reported swollen lymph nodes, no medical diagnosis reported). Of the remaining 13 players who completed POST_FALL_ testing, three did not return to the team in the spring and two did not complete POST_SPRING_ testing due to injury (*n* = 1) or non-compliance (*n* = 1). Consequently, eight players completed the cross-over portion of this study. This investigation was approved by the Kennesaw State University Institutional Review Board (#17–058).

### Supplementation intervention

Prior to enrollment, several potential participants had indicated that they regularly or periodically (i.e., only on training days) consumed creatine monohydrate as a dietary supplement. To standardize creatine monohydrate consumption, all enrolled athletes were given a 5-day supply (twenty 5 g packets for a total of 100 g) of creatine monohydrate in powder form (Bulk Supplements.com, Hard Eight Nutrition LLC, Henderson, NV). The athletes were instructed to consume four 5 g packets with water (375 mL) per day for 5 days to standardize muscle creatine content [10] and prevent changes to the athlete’s normal supplementation habits (totaling 20 g per day for 5-days). Subsequently, the athletes were divided into HMB-Cr (5 g HMB [Bulk Supplements.com, Hard Eight Nutrition LLC, Henderson, NV] + 5 g creatine monohydrate) or Cr (5 g creatine monohydrate + 5 g maltodextrin [Now Health Group Inc., Bloomingdale, IL]). Both supplements were similar in appearance, texture, and taste. Regardless of which supplementation regimen they were instructed to follow, the athletes ingested their given supplement, in powder form, with water (375 mL) once each day for two separate six-week periods. A member of the research team, who did not participate in data collection except to assist in obtaining blood samples, maintained the double blind (i.e., both remaining research team members and athletes did not know supplementation assignments) until after all data had been collected and analyzed. To aid in compliance, members of the research team met with participating athletes at practice on each week of the study to deliver a week’s worth of supplement and to collect used supplement packets. All athletes who respectively completed fall and spring testing returned all used supplement packets each week and did not report missing any dosages; indicating 100% compliance in those athletes.

### Blood sampling and biochemical analysis

Fasted blood samples were obtained on each visit to the HPL prior to any physical activity. All samples were obtained from an antecubital vein using a needle by a research team member who was trained and experienced in phlebotomy. Approximately 15 mL of blood was drawn into SST tubes (for serum collection) and EDTA-treated Vacutainer® tubes (for plasma). SST tubes were allowed to clot for 10 min prior to centrifugation, while EDTA treated tubes were centrifuged immediately for 10 min at 3600 rpms at 4 °C. The resulting serum and plasma were aliquoted and stored at − 80 °C until analysis.

All samples were analyzed for circulating concentrations of cortisol (ng·mL^− 1^) and creatine kinase (μ·L^− 1^). Concentrations of cortisol were analyzed via enzyme-linked immunosorbent assays (ELISA) via a 96-well spectrophotometer (SpectraMax M3 Multi-Mode Microplate Reader, Molecular Devices) using a commercially available kit. Concentrations of creatine kinase were determined against an enzymatic approach in serum samples using commercially available reagents and a single-cuvette spectrophotometer (SpectraMax M3 Multi-Mode Microplate Reader, Molecular Devices) at a wavelength of 340 and 450 nm (nm), respectively. To eliminate inter-assay variance, all samples were thawed once and analyzed in duplicate in the same run by a single technician with an average coefficient of variation of 4.2% for cortisol and 5.8% for creatine kinase.

### Body composition

Initially, height (± 0.1 cm) and body mass (± 0.1 kg) were determined using a stadiometer (WB-3000, TANITA Corporation, Tokyo, Japan) with the athletes standing barefoot, feet together, in athletic attire. Subsequently, body composition was assessed by three methods (i.e., dual energy X-ray absorptiometry [iDXA, Lunar Corporation, Madison, WI], air displacement plethysmography [BodPod, COSMED USA Inc., Chicago, IL], and bioelectrical impedance analysis [770 Body Composition and Body Water Analyzer, InBody, Seoul, South Korea]) using standardized procedures. Briefly, iDXA scanning required the athletes to remove any metal or jewelry and lay supine on the iDXA table prior to an entire body scan in “standard” mode using the company’s recommended procedures and supplied algorithms. Quality assurance was assessed by daily calibrations performed prior to all scans using a calibration block provided by the manufacturer. All iDXA measurements were performed by the same researcher using standardized positioning procedures. For air displacement plethysmography, the device and associated scale were calibrated daily using a known volume and mass provided by the manufacturer. During testing, the athletes were asked to wear a tight-fitting bathing suit or compression shorts and swim cap before entering the device. Two trials were performed for each athlete to obtain two measurements of body volume within 150 mL. A third trial was performed if body volume estimates from the first two trials were not within 150 mL, and values from the two closest trials were averaged. Thoracic lung volume was estimated [36]. Bioelectrical impedance analysis required the athletes to stand barefoot on two metal sensors located at the base of the device and hold two hand grips for approximately 30–60 s. Prior to stepping onto the device, the athletes cleaned the soles of their feet with alcohol wipes provided by the manufacturer.

Following testing, body mass, bone mineral content (BMC; from iDXA), body volume (from BodPod), and total body water (from bioelectrical impedance analysis) were entered into a 4-compartment model (Eq. 1), to estimate body fat percentage (BF%) [37] and fat-free mass (± 0.1 kg).

1 $$ BF\%=\frac{\left(2.748\ x\  volume\right)-\left(0.699\ x\  water\right)+\left(1.129\ x\  BMC\right)-\left(2.051\ x\  Body\ Mass\right)}{Body\ Mass}x\ 100 $$

### Muscle morphology

Non-invasive skeletal muscle ultrasound images were collected from the right thigh of each athlete. Prior to image collection, all anatomical locations of interest were identified using standardized landmarks for the rectus femoris (RF; 50% of the distance from the proximal border of the patella to the anterior, inferior suprailiac crest) and vastus lateralis (VL; 50% of the distance from the lateral condyle of the tibia to the most prominent point of the greater trochanter of the femur) muscles. After completion of all measurements, the athlete laid supine on the examination table for a minimum of 15 min to allow fluid shifts to occur before images were collected [38]. The same investigator performed all landmark measurements for each participant.

A 12 MHz linear probe scanning head (General Electric Vivid i BT09, Wauwatosa, WI, USA) was coated with water soluble transmission gel to optimize spatial resolution and used to collect all ultrasound images. Image collection began with the probe being positioned on (and perpendicular to) the surface of the skin to provide acoustic contact without depressing the dermal layer. Subsequently, two consecutive images were collected with the probe oriented longitudinal to the muscle tissue interface using Brightness Mode (B-mode; Gain = 50 dB; image depth = 5–6 cm) ultrasound [39]. Each of these images included a horizontal line (approximately 1 cm), located below the image, which was used for calibration purposes when analyzing the images offline [40]. To capture images of the RF, the athlete laid supine with his legs extended but relaxed. A rolled towel was placed beneath the popliteal fossa of the right leg to allow a 10° bend in the knee and the foot was secured [41]. For the VL, the athlete was placed on his side, legs together except for a rolled towel between the knees, and positioned to allow a 10° bend at the knees [41]. The same investigator positioned each athlete and collected all images.

After all images were collected, the ultrasound data were transferred to a personal computer for analysis via Image J (National Institutes of Health, Bethesda, MD, USA, version 1.45 s) by the same technician. Muscle thickness (± 0.01 cm; perpendicular distance between the superficial and deep aponeuroses) and pennation angle (± 0.1°; intersection of the fascicles with the deep aponeurosis) were measured from each image and averaged. Intraclass correlation coefficients (ICC_3,k_ = 0.77–0.99) for determining muscle thickness (RF: ICC_3,K_ = 0.93, VL: ICC_3,K_ = 0.88) and pennation angle (RF: ICC_3,K_ = 0.99, VL: ICC_3,K_ = 0.84) was previously determined in ten active, resistance-trained men (25.3 ± 2.0 years, 180 ± 7 cm, 90.8 ± 6.8 kg) using the methodology described above.

### Maximal and rapid torque production

Following anthropometric assessments, the athletes completed a general warm-up that included riding a cycle ergometer for 5 min at their preferred resistance and cadence followed by a dynamic stretching: 10 body weight squats, 10 alternating lunges, 10 walking knee hugs and 10 walking butt kicks. The athletes then completed three maximal voluntary isometric contractions (MVICs) of the right knee extensors and flexors on a Biodex System 4 dynamometer (Biodex Medical Systems, Inc. Shirley, NY, USA) at a knee joint angle of 120° and 150° (180° = full knee extension), respectively [42, 43]. The order of testing (e.g., knee flexion before knee extension) was randomized at PRE_FALL_ and remained constant for each athlete on subsequent visits. Athletes were seated on the dynamometer with their hands across their chest and restraining straps placed over their trunk, pelvis, and thigh. The axis of knee rotation was aligned with the input axis of the dynamometer. Initially, athletes performed two submaximal isometric contractions at 50 and 75% of their perceived maximal effort prior to maximal testing. For maximal testing, the athletes were instructed to “push” or “pull”, “as hard and fast as possible” for 3–4 s for the knee extensors and knee flexors, respectively [44, 45]. A one-minute rest interval separated each trial. Athletes were instructed to keep the leg musculature relaxed and to avoid a countermovement prior to each MVIC. An additional trial was performed if any preceding activity occurred during baseline. Strong verbal encouragement and visual biofeedback was provided during all maximal strength tests. The dynamometer chair settings were recorded to ensure that identical settings were used for each subsequent visit.

The raw torque data from the dynamometer was processed using custom written software (LabVIEW, National Instruments, Austin, TX, USA). Peak torque (Nm) was considered the highest 50 ms rolling average. Rate of torque development (RTD; Nm·sec^− 1^) was derived from the linear slope of the torque-time curve (Δtorque / Δtime) and calculated from 0 to 100 ms and 0–200 ms. Impulse (Nm·sec) was calculated as the area under the torque-time curve (∫Torque *dt*) for the same time intervals. A torque onset of 7.5 Nm and 4 Nm was used for the knee extensors and knee flexors, respectively [45]. The trial producing the highest peak torque was used for subsequent analysis.

### Sprinting performance

Sprinting time and kinetics (i.e., force, velocity, and power) were assessed during 40-m sprints using an isokinetic sprinting device (1080 Sprint, 1080 Motion, Lidingo, Sweden) tethered to athletes via waist belt (Power Systems LLC, Knoxville, TN, USA). All sprints were performed in cleats on a grass surface located adjacent to their training facility. A pair of cones were positioned approximately 5 m from the device to denote the “starting line”, while another pair of cones were placed parallel to the starting line at a distance of 40 m to denote the “finish line”. Three additional sets of cones were placed approximately 10 m apart between the starting and finish lines to clearly indicate the sprinting path. Prior to testing, the athletes performed their usual pre-practice warm-up routine, which included approximately 5 min of light jogging followed by dynamic stretching. Then the athletes completed a specific warm-up that included two untethered submaximal sprints and one maximal familiarization 40-m sprint while tethered to the device. Subsequently, they completed one maximal sprint trial against the low-resistance (9.81 N; the minimum resistance necessary for the device to measure kinetics) and a second against high-resistance (147.1 N; the maximal resistance setting). The athletes were allotted 3–5 min of rest between each sprint. All sprint tests were conducted in the “Isotonic” mode (i.e., load is constant and independent of acceleration) to provide the smoothest resistance during testing, as per the manufacturer recommendations. The athletes were instructed to take their preferred starting stance at the starting line and to begin each sprint at their ready. The Quantum software used to control the device was set to detect the athlete’s position and initiate data collection on his first movement and continue to collect data until the athlete reached a distance of 40 m from his starting position. Athletes were verbally encouraged throughout each maximal sprint. The starting leg was recorded during the first sprint and made constant throughout all future sprints. Upon completion of each testing session, sprinting data were stored on a password-protected computer for subsequent analysis.

Sprinting kinetic data were downloaded from the Quantum software into a comma-separated values file and organized into a spreadsheet (Excel 2016, Microsoft, Redmond, WA, USA). Data from each file was used to calculate sprinting kinetic variables as previously described [46]. Briefly, changes in acceleration were used to identify the onset and duration of each step and then force (N), velocity (m·sec^− 1^), and power (W) were averaged for each step. Calculated values were then separated into the first and second steps, the acceleration phase (i.e., from the 3rd step to the step when peak velocity was achieved), and the peak velocity phase (i.e., the remainder of the sprint). Since the acceleration phase is marked by increases in velocity, the rate of increase for each kinetic variable was used for all group comparisons. In contrast, the average across all steps in the peak velocity phase was used for group comparisons due to the consistency seen in velocity during this phase. The repeatability (± 0.7%) and accuracy of the 1080 Sprint for measuring position (± 0.5%), velocity (± 0.5%) and force (± 4.8 N) have been provided by the manufacturer [47], while we have also demonstrated consistent measures of kinetics following two consecutive sprints [46].

### Dietary intake and training

Due to the known influence on muscle size and performance, athletes were instructed to maintain their normal kilocaloric intake and training habits throughout the course of the investigation. The athletes were asked to record all food and beverage intake over the course of 3 days (two weekdays and one weekend day) on two consecutive weeks at the onset (i.e., weeks 1 and 2) and conclusion (i.e., weeks 5 and 6) of the fall and spring competitive seasons. Food records were collected weekly and reviewed by a research team member before entering the information into a publicly-available online database (MyFitnessPal) to determine total kilocaloric (kcals) and protein (g) intake. For statistical analysis, average kilocaloric and protein intake over each consecutive 2-wk period were analyzed relative to body mass.

Records of the occurrence and details of resistance training were also requested of athletes because the team did not utilize a standard training program. The athletes were asked to provide a list of the exercises they completed, as well as the number of sets, repetitions, intensity load, and approximate rest intervals that they used for each session. These records were collected on two consecutive weeks at the onset and conclusion of each season. Since each athlete utilized his own training regimen, there was considerable variability in the number of training days completed each week, exercises performed, set and repetition paradigms, and whether rest intervals were tracked. Briefly, the athletes reported participating in training sessions on 1–7 days each week, they utilized a various assortment of structural (e.g., bench press, shoulder press, squat, deadlift), core (e.g., lat pulldowns, seated rows, upright rows), and assistance exercises (e.g., biceps curls, triceps pushdowns) with barbells, dumbbells, and cables. The number of sets and repetitions completed varied considerably between typical muscular strength, hypertrophy, and endurance paradigms; though each athlete remained fairly consistent with their own training pattern. To quantify training, frequency (days·wk.^− 1^) and upper- and lower-body volume (sets·repetitions·load) were averaged over each 2-week period at the beginning and end of each season and then used for statistical comparisons.

### Statistical analysis

The Shapiro-Wilk test was used to determine normality and indicated that several of the variables were not distributed normally. Consequently, changes in all variables were separately examined across time using linear mixed models with maximum likelihood estimation and an autoregressive-heterogenous repeated covariance to account for the dependent relationships existing between time points. Additionally, a grouping factor (i.e., HMB-Cr or Cr) was added to the model to examine the effect of supplementation. Following any significant F-ratio, specific differences between time points within each group were further assessed by applying Bonferonni adjustments to confidence intervals while differences between groups at each time point were examined using independent *t*-tests. For all statistical tests, a criterion alpha level of *p* ≤ 0.05 was used to determine significance. Differences between and within groups were further assessed by effect sizes calculated according to Cohen’s d and Cohen’s dz., respectively [48]. As previously suggested for recreationally-trained individuals [49], interpretations of effect size were evaluated at the following levels: trivial (< 0.35), small (0.35–0.80), moderate (0.80–1.50), and large (> 1.50). Data are represented as mean (or mean difference) ± standard deviation with 95% confidence intervals (95% C.I.).

## Results

Over the course of the entire season, the athletes participated in two training sessions per week and 8 total matches. The fall season consisted of 3 matches and the athletes averaged 16.5 out of 24 total practice hours (68.7% of total practice hours or 1.37 h per practice) and 174 min of gameplay (~ 58 min per match) across the season. During the spring season, there were 5 matches and the athletes averaged 27 out of 32 total practice hours (84.3% of total practice hours or 1.69 h per practice) and 280 min of gameplay (~ 56 min per match). No differences were seen between groups in practice or gameplay hours during either the fall or spring seasons. No practices were held between the fall and spring seasons.

### Biochemical analyses

**Fig. 2:**
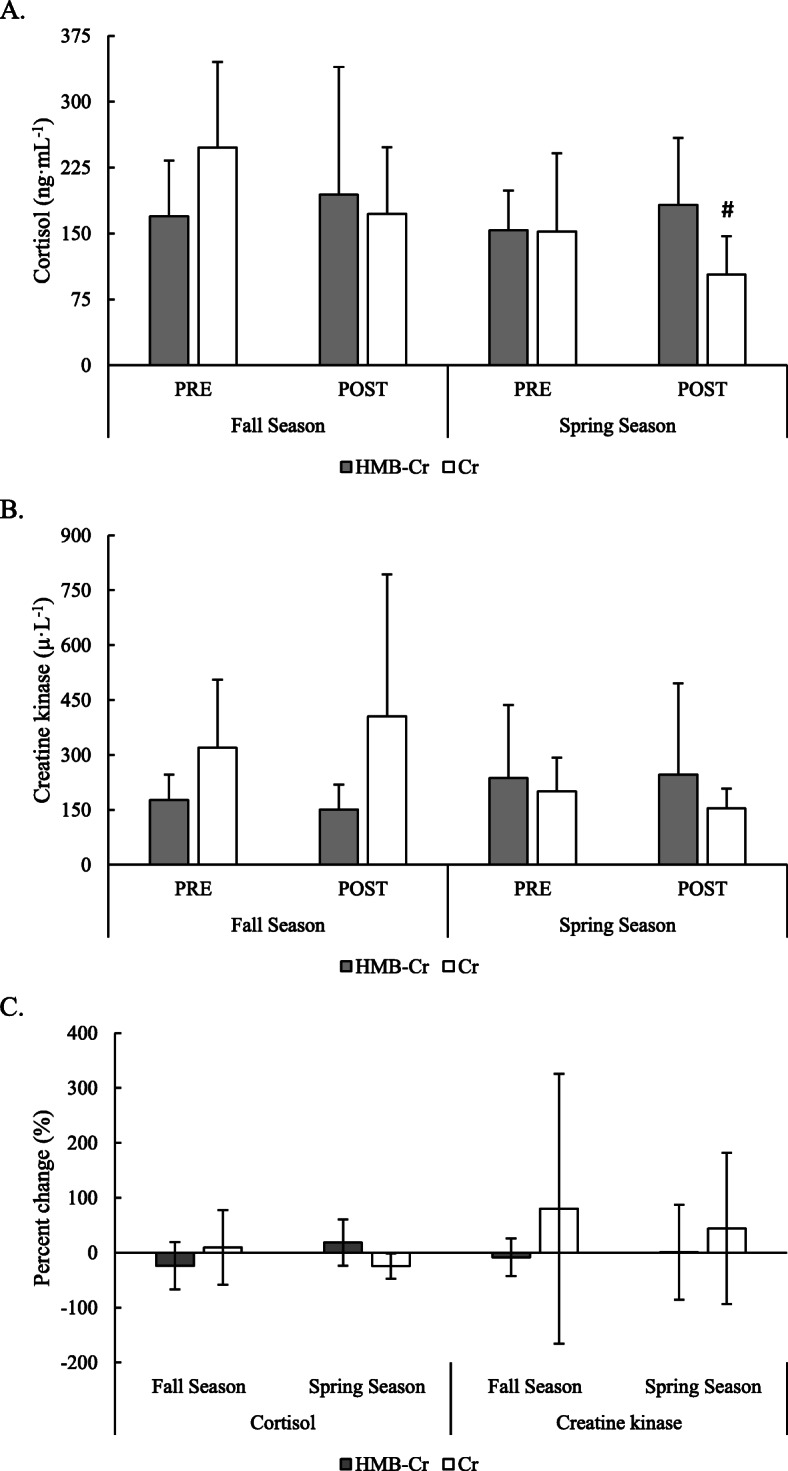
Changes in circulating concentrations of **a**) cortisol and **b**) creatine kinase over the course of the fall and spring rugby seasons. # = Different (*p* <  0.10) from PRE_FALL_

A significant group x time interaction was observed for concentrations of cortisol (*F* = 3.76, *p* = 0.030). However, post-hoc analysis only revealed a tendency for concentrations to be lower by approximately 58.3% (mean difference = 144.5 ng·mL^− 1^; 95% C.I. = − 290 to 0.9 ng·mL^− 1^) in Cr at POST_SPRING_ compared to PRE_FALL_ concentrations (*p* = 0.052, *d* = 1.35);. Though no significant interaction was observed for concentrations of creatine kinase, a trend for a group x time interaction (*F* = 2.63, *p* = 0.085) indicated a time effect for Cr (*p* = 0.061), but specific differences between time points were not found. Changes in concentrations of cortisol and creatine kinase are illustrated in Fig. 2.

### Body composition and muscle morphology

Although a group x time interaction was observed for VL pennation angle, post-hoc analysis only revealed a tendency for pennation angle to be shorter by approximately 2.55° (95% C.I. = − 5.22 to 0.12°) in Cr at PRE_SPRING_ compared to PRE_FALL_ (*p* = 0.066, *d* = 1.20). Otherwise, no other significant changes or group differences were observed for any other measure of body composition or muscle morphology. Changes in measures of body composition and muscle morphology are presented in Table 1.

**Table 1:** Changes in body composition and muscle morphology over the course of the fall and spring rugby seasons

	Fall Season		Spring Season		Group x Time	
	PRE	POST	PRE	POST	F	***p***-value
Body composition						
Height (cm)						
HMB-Cr	177 ± 7 (172 to 181)	176 ± 8 (172 to 181)	179 ± 5 (174 to 184)	178 ± 5 (175 to 185)	0.55	0.651
Cr	179 ± 6 (175 to 184)	179 ± 5 (174 to 184)	179 ± 8 (173 to 183)	182 ± 4 (173 to 183)		
*d*	0.37	0.46	0.01	0.85		
Body mass (kg)						
HMB-Cr	88.4 ± 15.3 (78.1 to 98.7)	88.2 ± 16.3 (78.3 to 98.5)	93.5 ± 14.7 (79.1 to 99.3)	95.4 ± 16.9 (79.6 to 99.2)	0.03	0.992
Cr	88.3 ± 14.1 (78.0 to 98.6)	89.0 ± 14.5 (78.4 to 98.5)	90.6 ± 16.9 (77.9 to 98.0)	97.6 ± 11.1 (78.9 to 98.3)		
*d*	0.01	0.05	0.19	0.17		
Body fat percentage (%)						
HMB-Cr	15.1 ± 6.5 (10.4 to 19.7)	13.3 ± 6.3 (8.6 to 17.2)	18.9 ± 7.5 (7.1 to 20.4)	21.6 ± 6.0 (9.2 to 21.4)	0.47	0.709
Cr	14.8 ± 6.8 (10.1 to 19.4)	13.0 ± 6.7 (7.4 to 16.1)	14.8 ± 11.0 (7.0 to 19.9)	18.9 ± 7.9 (7.5 to 19.2)		
*d*	0.05	0.05	0.44	0.40		
Fat-free mass (kg)						
HMB-Cr	74.5 ± 10.1 (68.1 to 80.9)	75.9 ± 10.6 (69.7 to 83.2)	75.1 ± 6.5 (68.0 to 82.0)	74.2 ± 8.3 (67.8 to 81.8)	0.40	0.755
Cr	74.7 ± 8.1 (68.3 to 81.1)	76.8 ± 7.7 (70.9 to 84.5)	76.1 ± 11.0 (68.2 to 81.6)	78.5 ± 4.3 (70.2 to 83.4)		
*d*	0.02	0.10	0.11	0.70		
Muscle thickness (cm)						
Rectus femoris						
HMB-Cr	2.73 ± 0.50 (2.40 to 3.06)	2.68 ± 0.47 (2.36 to 3.02)	2.90 ± 0.12 (2.63 to 3.15)	15.78 ± 3.56 (2.52 to 3.42)	0.60	0.629
Cr	2.92 ± 0.36 (2.59 to 3.25)	2.75 ± 0.35 (2.44 to 3.15)	2.84 ± 0.37 (2.60 to 3.03)	8.90 ± 6.69 (2.67 to 3.45)		
*d*	0.44	0.15	0.21	1.09		
Vastus lateralis						
HMB-Cr	1.89 ± 0.33 (1.64 to 2.14)	2.11 ± 0.28 (1.80 to 2.36)	2.32 ± 0.28 (1.92 to 2.46)	2.06 ± 0.18 (1.81 to 2.27)	1.77	0.194
Cr	2.03 ± 0.44 (1.78 to 2.28)	2.11 ± 0.52 (1.80 to 2.38)	1.81 ± 0.21 (1.55 to 2.03)	1.82 ± 0.14 (1.63 to 2.03)		
*d*	0.38	< 0.01	1.48	1.24		
Pennation angle (°)						
Rectus femoris						
HMB-Cr	16.2 ± 2.7 (13.7 to 18.8)	15.7 ± 4.9 (12.7 to 18.8)	14.6 ± 3.8 (11.7 to 17.3)	20.2 ± 5.3 (15.2 to 25.5)	0.90	0.469
Cr	15.5 ± 4.3 (12.9 to 18.0)	15.7 ± 2.6 (12.4 to 19.0)	14.3 ± 2.0 (12.0 to 16.6)	14.8 ± 4.1 (10.5 to 19.4)		
*d*	0.22	< 0.01	0.11	1.06		
Vastus lateralis						
HMB-Cr	12.8 ± 2.2 (11.6 to 14.1)	12.8 ± 2.1 (11.2 to 14.6)	16.3 ± 2.2 (14.2 to 18.6)	14.4 ± 3.1 (11.7 to 17.0)	5.75	0.008
Cr	14.5 ± 1.4 (13.2 to 15.8)	13.2 ± 2.4 (11.4 to 15.0)	12.0 ± 2.2 (10.2 to 13.7)#	13.0 ± 1.6 (10.6 to 15.3)		
*d*	0.85	0.15	1.43	0.62		

# = Difference (*p* < 0.10) from PRE_FALL_

### Maximal and rapid torque production

A significant group x time interaction was observed for knee extension peak torque which decreased from PRE_FALL_ to POST_FALL_ (mean difference = − 40.7 ± 28.1 Nm, 95% C.I. = − 78.5 to − 2.85 Nm, *p* = 0.035, *dz* = − 1.45) for HMB-Cr only. Significant group x time interactions were also found for knee flexion peak torque, RTD from 0 to 200 ms, and impulse (0–100 ms and 0–200 ms). Although post hoc analysis did not reveal any significant changes for either group, RTD at 0–200 ms was greater (*p* = 0.003, *d* = 1.73) in Cr by approximately 149 Nm·sec^− 1^ (95% C.I. = 80 to 218 Nm·sec^− 1^, *p* = 0.003) and impulse at 0–200 ms was greater (*p* = 0.022, *d* = 1.54) in Cr by approximately 4.5 Nm·sec (95% C.I. = 0.9 to 8.0 Nm·sec) at PRE_SPRING_. No other specific group differences or changes were observed. Changes in isometric knee extensor and flexor maximal and rapid torque production are presented in Table 2.

**Table 2:** Changes in maximal and rapid torque production of the knee extensors and flexors over the course of the fall and spring rugby seasons

	Fall Season		Spring Season		Group x Time	
	PRE	POST	PRE	POST	F	***p***
Knee Extension						
Peak Torque (Nm)						
HMB-Cr	293 ± 57 (255 to 331)	253 ± 61 (213 to 292)*	294 ± 39 (224 to 342)	331 ± 42 (254 to 389)	7.31	0.005
Cr	301 ± 55 (264 to 339)	292 ± 61 (253 to 333)	314 ± 30 (223 to 336)	268 ± 33 (175 to 300)		
*d*	0.15	0.63	0.59	1.33		
RTD_0–100_ (Nm·sec^−1^)						
HMB-Cr	2073 ± 498 (1742 to 2405)	1512 ± 456 (1187 to 1857)	1689 ± 334 (1410 to 1988)	1770 ± 538 (1275 to 2282)	1.99	0.159
Cr	1935 ± 449 (1603 to 2267)	1661 ± 537 (1307 to 2014)	2135 ± 155 (1784 to 2296)	1812 ± 283 (1285 to 2164)		
*d*	0.3	0.31	1.37	0.11		
RTD_0–200_ (Nm·sec^−1^)						
HMB-Cr	1290 ± 255 (1131 to 1449)	873 ± 198 (701 to 1093)	1075 ± 203 (874 to 1392)	1135 ± 329 (783 to 1598)	2.59	0.105
Cr	1195 ± 213 (1036 to 1354)	1012 ± 365 (810 to 1220)	1305 ± 155 (1019 to 1485)	1080 ± 298 (671 to 1388)		
*d*	0.41	0.49	1.14	0.19		
Impulse_0–100_ (Nm·sec)						
HMB-Cr	10.4 ± 2.4 (8.9 to 12.0)	8.1 ± 2.1 (6.7 to 9.4)	8.2 ± 1.5 (6.8 to 9.7)	8.1 ± 1.7 (6.4 to 9.8)	2.13	0.146
Cr	9.2 ± 2.0 (7.7 to 10.8)	8.6 ± 1.6 (7.2 to 10.0)	10.5 ± 0.9 (8.9 to 11.5)	8.7 ± 0.9 (7.2 to 10.1)		
*d*	0.54	0.29	1.39	0.57		
Impulse_0–200_ (Nm·sec)						
HMB-Cr	34.2 ± 6.8 (30.0 to 38.4)	25.1 ± 5.7 (21.0 to 29.3)	27.2 ± 5.0 (22.8 to 32.6)	27.5 ± 6.2 (20.4 to 35.2)	2.67	0.097
Cr	30.6 ± 5.5 (26.3 to 34.8)	27.3 ± 6.5 (22.9 to 31.8)	34.2 ± 2.8 (28.6 to 37.3)	28.5 ± 4.9 (21.3 to 34.1)		
*d*	0.58	0.38	1.36	0.20		
Knee Flexion						
Peak Torque (Nm)						
HMB-Cr	177 ± 34 (156 to 199)	172 ± 48 (145 to 196)	186 ± 10 (156 to 201)	198 ± 15 (156 to 221)	4.40	0.025
Cr	174 ± 27 (151 to 197)	189 ± 20 (159 to 214)	200 ± 10 (158 to 200)	194 ± 17 (138 to 197)		
*d*	0.12	0.44	1.21	0.24		
RTD_0–100_ (Nm·sec^−1^)						
HMB-Cr	1149 ± 266 (948 to 1350)	1040 ± 261 (838 to 1215)	876 ± 162 (672 to 1103)	1026 ± 280 (413 to 1677)	2.90	0.094
Cr	971 ± 361 (756 to 1185)	1076 ± 354 (826 to 1251)	1240 ± 159 (949 to 1327)	1144 ± 373 (415 to 1517)		
*d*	0.57	0.13	1.49	0.37		
RTD_0–200_ (Nm·sec^−1^)						
HMB-Cr	750 ± 132 (659 to 841)	704 ± 204 (569 to 812)	706 ± 35 (653 to 741)	755 ± 83 (554 to 902)	4.91	0.014
Cr	732 ± 136 (635 to 830)	737 ± 143 (574 to 843)	855 ± 35 (781 to 859)†	767 ± 160 (513 to 819)		
*d*	0.14	0.19	1.73	0.10		
Impulse_0–100_ (Nm·sec)						
HMB-Cr	5.1 ± 1.3 (4.3 to 6.0)	4.7 ± 0.8 (3.7 to 5.6)	3.9 ± 0.9 (3.0 to 5.3)	4.3 ± 0.8 (2.1 to 7.1)	4.19	0.040
Cr	3.9 ± 1.3 (3.0 to 4.8)	4.9 ± 2.1 (3.7 to 5.8)	5.3 ± 0.7 (3.9 to 6.0)	5.3 ± 1.7 (2.7 to 7.0)		
*d*	0.89	0.16	1.36	0.74		
Impulse_0–200_ (Nm·sec)						
HMB-Cr	18.0 ± 3.7 (15.5 to 20.6)	16.7 ± 3.6 (13.9 to 19.0)	15.2 ± 2.0 (12.5 to 18.4)	16.5 ± 1.4 (9.5 to 24.7)	4.92	0.023
Cr	15.4 ± 3.8 (12.7 to 18.1)	17.5 ± 4.7 (14.1 to 19.8)	19.6 ± 1.6 (14.9 to 20.2)†	18.5 ± 5.0 (7.8 to 21.4)		
*d*	0.67	0.19	1.54	0.53		

* = Significant (*p* < 0.05) difference from PRE_FALL_; † = Significant (*p* < 0.05) difference between groups

### Sprinting performance

No differences in sprint time were noted during the low-resistance sprint for HMB-Cr (PRE_FALL_: 6.00 ± 0.28 s; POST_FALL_: 5.90 ± 0.47 s; PRE_SPRING_: 6.05 ± 0.41 s; POST_SPRING_: 6.09 ± 0.63 s) and Cr (PRE_FALL_: 6.06 ± 0.35 s; POST_FALL_: 6.09 ± 0.26 s; PRE_SPRING_: 6.10 ± 0.37 s; POST_SPRING_: 6.08 ± 0.21 s) over the course of the study. Likewise, HMB-Cr (PRE_FALL_: 8.32 ± 0.76 s; POST_FALL_: 8.07 ± 0.94 s; PRE_SPRING_: 8.03 ± 0.69 s; POST_SPRING_: 8.03 ± 0.93 s) and Cr (PRE_FALL_: 8.17 ± 0.89 s; POST_FALL_: 8.35 ± 0.81 s; PRE_SPRING_: 8.47 ± 1.11 s; POST_SPRING_: 8.33 ± 0.66 s) produced similar sprint times during the high-resistance sprint. Additionally, each group averaged the same number of steps, step distances, and step durations for each sprinting phase across each season.

**Fig. 3:**
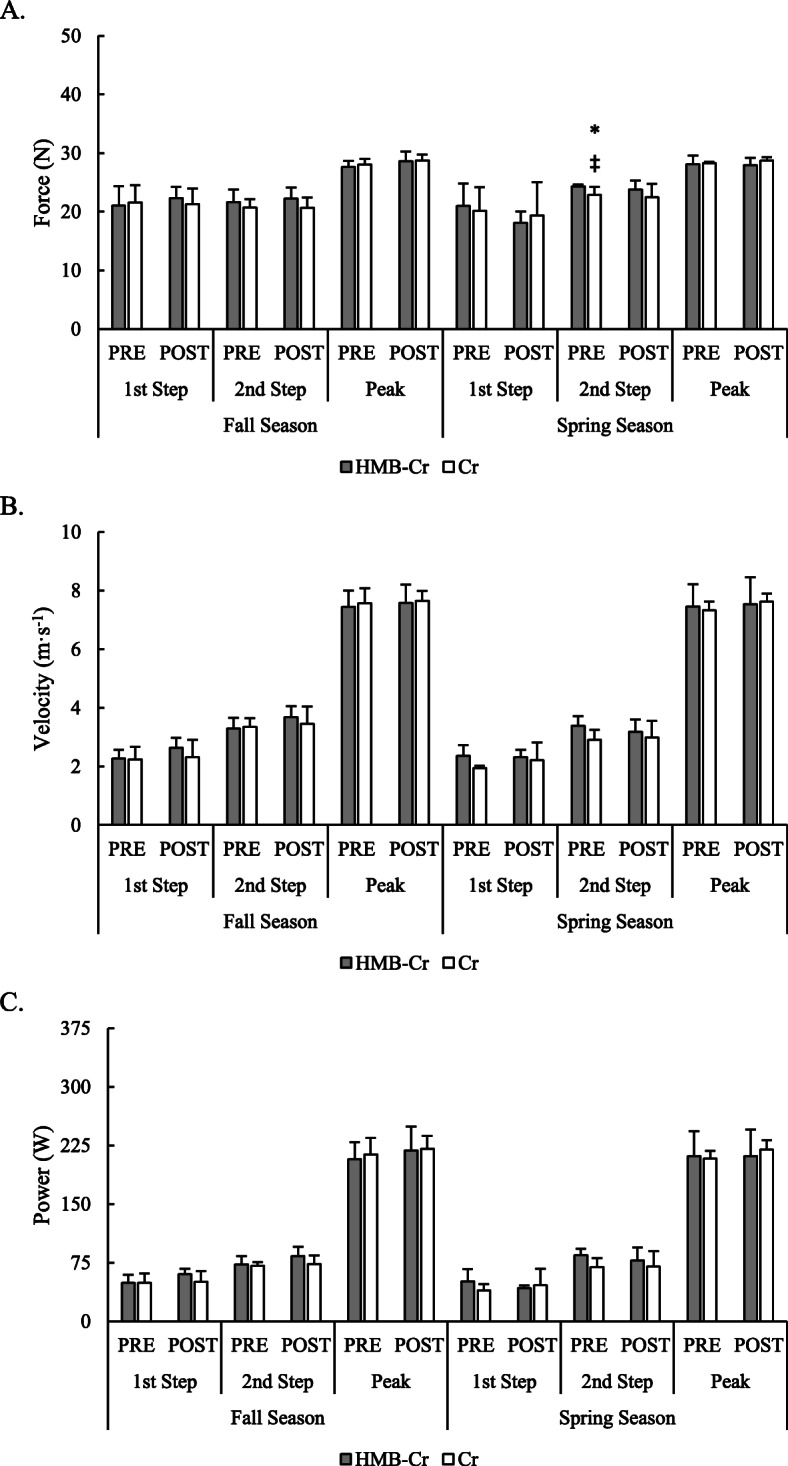
Changes in kinetics during a low-resistance sprint over the course of the fall and spring rugby seasons. * = Significantly (*p* <  0.05) different from PRE_FALL_; ‡ = Significantly (*p* <  0.05) different from POST_FALL_

**Fig. 4:**
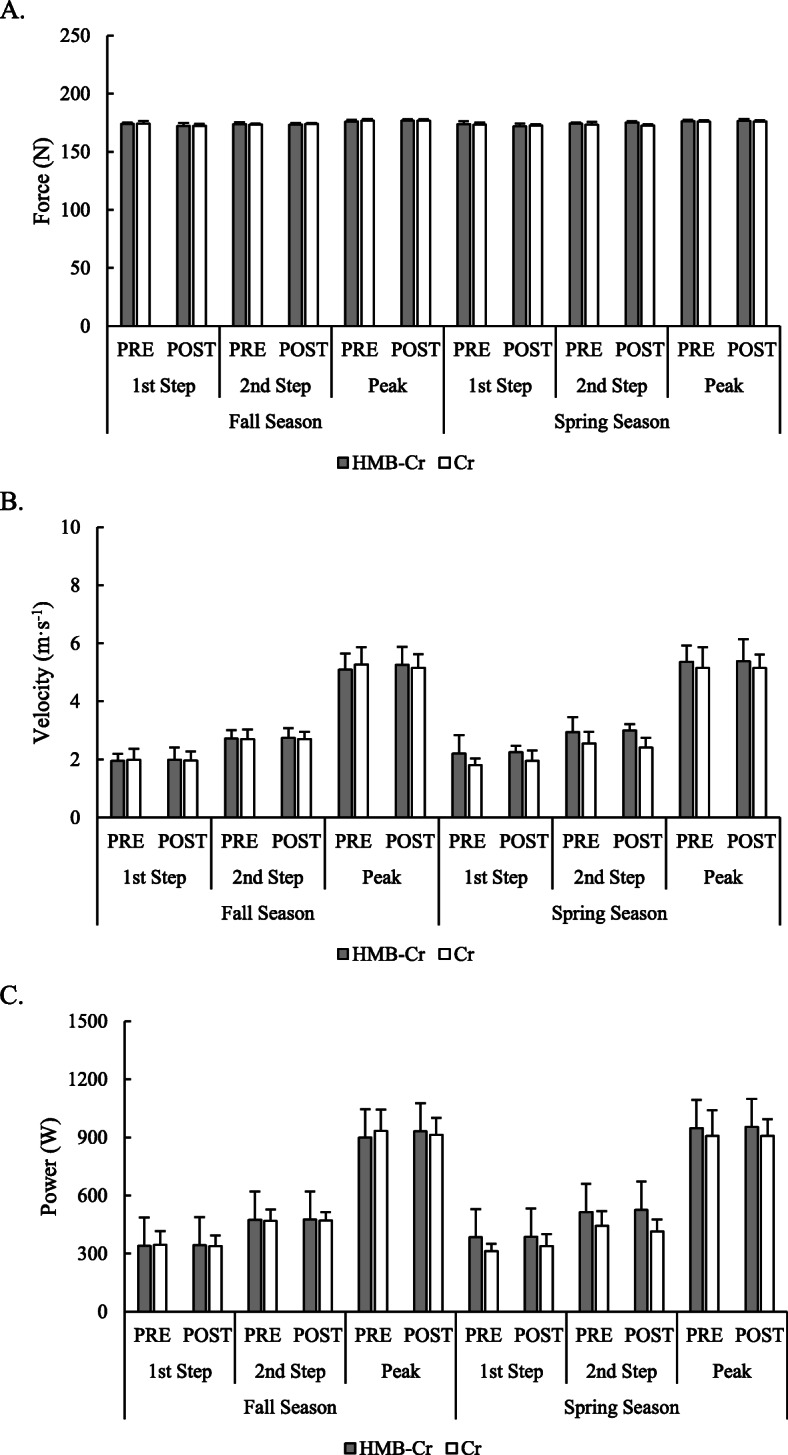
Changes in kinetics during a high-resistance sprint over the course of the fall and spring rugby seasons

A significant group x time interaction was observed on the second step of the low-resistance sprint for force (*F* = 2.67, *p* = 0.037). At PRE_SPRING_, force was reduced for Cr only by approximately 3.61 N (95% C.I. = − 1.41 to – 5.81 N) compared to PRE_FALL_ (*p* = 0.002, *d* = 1.49) and by approximately 3.60 N (95% C.I. = − 0.23 to − 6.97 N) compared to POST_FALL_ (*p* = 0.035, *d* = 1.03). An interaction was also noted for power on the second step (*F* = 3.29, *p* = 0.028) but only expected differences between resistance intensities (i.e., between low-resistance and high-resistance) were revealed by post hoc analysis. No other differences were found between groups over time. Sprinting kinetics produced on the first two steps and averaged across the peak velocity phase during the low-resistance and high-resistance sprints are illustrated in Figs. 3 and 4, respectively, while changes in kinetics during the acceleration phase of both sprints are presented in Table 3.

**Table 3:** Rate of change in kinetics during acceleration phase of 40-m sprint against low- and high-resistance over the course of the fall and spring rugby seasons

		Fall Season		Spring Season		Group x Time	
		Pre	Post	Pre	Post	F	***p***
Average Force rate (N·step^−1^)							
Low-resistance	HMB-Cr	0.32 ± 0.20 (0.19 to 0.45)	0.30 ± 0.07 (0.18 to 0.42)	0.20 ± 0.08 (0.12 to 0.28)	0.22 ± 0.13 (0.07 to 0.32)	0.47	0.843
	Cr	0.35 ± 0.15 (0.23 to 0.47)	0.34 ± 0.17 (0.23 to 0.447)	0.20 ± 0.05 (0.13 to 0.26)	0.20 ± 0.05 (0.11 to 0.33)		
	*d*	0.17	0.29	0.11	0.23		
High-resistance	HMB-Cr	0.18 ± 0.16 (0.07 to 0.30)	0.26 ± 0.11 (0.13 to 0.39)	0.18 ± 0.09 (0.06 to 0.23)	0.14 ± 0.05 (0.08 to 0.21)		
	Cr	0.30 ± 0.13 (0.20 to 0.40)	0.31 ± 0.18 (0.17 to 0.44)	0.14 ± 0.03 (0.11 to 0.24)	0.15 ± 0.06 (0.08 to 0.19)		
	*d*	0.78	0.33	0.54	0.19		
Average Velocity rate (m·sec^−1^·step^−1^)							
Low-resistance	HMB-Cr	0.27 ± 0.11 (0.21 to 0.33)	0.25 ± 0.08 (0.19 to 0.32)	0.20 ± 0.07 (0.13 to 0.28)	0.24 ± 0.12 (0.08 to 0.29)	0.84	0.569
	Cr	0.22 ± 0.05 (0.17 to 0.28)	0.19 ± 0.07 (0.13 to 0.25)	0.21 ± 0.04 (0.15 to 0.26)	0.18 ± 0.03 (0.14 to 0.32)		
	d	0.56	0.80	0.01	0.60		
High-resistance	HMB-Cr	0.15 ± 0.02 (0.13 to 0.17)	0.17 ± 0.05 (0.13 to 0.22)	0.13 ± 0.03 (0.09 to 0.15)	0.13 ± 0.05 (0.09 to 0.19)		
	Cr	0.14 ± 0.04 (0.12 to 0.16)	0.13 ± 0.05 (0.08 to 0.17)	0.12 ± 0.02 (0.11 to 0.16)	0.14 ± 0.03 (0.09 to 0.17)		
	*d*	0.27	0.91	0.34	0.25		
Average Power rate (W·step^−1^)							
Low-resistance	HMB-Cr	9.4 ± 4.0 (7.0 to 11.6)	8.7 ± 2.6 (6.3 to 11.1)	6.9 ± 2.7 (4.0 to 9.4)	7.9 ± 4.6 (2.2 to 10.2)	0.52	0.809
	Cr	8.2 ± 2.1 (6.0 to 10.3)	7.1 ± 2.8 (4.8 to 9.1)	6.8 ± 1.0 (4.81 to 9.1)	6.2 ± 0.8 (4.3 to 11.2)		
	*d*	0.39	0.60	0.05	0.51		
High-resistance	HMB-Cr	27.1 ± 4.3 (22.6 to 31.3)	31.8 ± 8.2 (23.2 to 40.2)	24.2 ± 6.0 (16.1 to 28.2)	24.1 ± 8.7 (16.7 to 34.6)		
	Cr	26.1 ± 7.3 (22.1 to 30.2)	24.0 ± 8.8 (15.3 to 32.4)	22.2 ± 4.3 (19.6 to 29.0)	25.6 ± 6.0 (16.3 to 31.7)		
	*d*	0.17	0.87	0.39	0.22		

### Dietary intake and training

Although a significant group x time interaction was observed (*F* = 5.19, *p* = 0.026), post hoc analysis did not reveal any significant changes or group differences in relative caloric intake for HMB-Cr (PRE_FALL_: 58.6 ± 12.5 kCal·kg^− 1^; POST_FALL_: 29.6 ± 11.2 kCal·kg^− 1^; PRE_SPRING_: 75.3 ± 16.2 kCal·kg^− 1^; POST_SPRING_: 83.0 ± 15.0 kCal·kg^− 1^) or Cr (PRE_FALL_: 60.6 ± 11.7 kCal·kg^− 1^; POST_FALL_: 70.2 ± 9.5 kCal·kg^− 1^; PRE_SPRING_: 86.0 ± 17.2 kCal·kg^− 1^; POST_SPRING_: 63.5 ± 17.1 kCal·kg^− 1^). Similarly, relative protein intake also remained consistent (*F* = 1.50, *p* = 0.262) for HMB-Cr (PRE_FALL_: 2.8 ± 0.8 g·kg^− 1^; POST_FALL_: 1.8 ± 1.0 g·kg^− 1^; PRE_SPRING_: 2.4 ± 0.4 g·kg^− 1^; POST_SPRING_: 3.5 ± 0.6 g·kg^− 1^) and Cr (PRE_FALL_: 3.6 ± 0.8 g·kg^− 1^; POST_FALL_: 4.1 ± 0.8 g·kg^− 1^; PRE_SPRING_: 2.2 ± 0.4 g·kg^− 1^; POST_SPRING_: 2.4 ± 0.7 g·kg^− 1^). Additionally, no differences between groups for training frequency or volume over the course of the study.

## Discussion

The primary aim of this investigation was to examine the effect of adding HMB to creatine monohydrate supplementation on changes in markers of biological stress and damage, body composition, muscle morphology, strength and sprinting performance across a rugby season. We hypothesized that the addition of HMB would attenuate markers of biological stress and damage, and better maintain body composition and performance over the course of the season, compared to creatine monohydrate supplementation alone. However, our data did not suggest the addition of HMB to creatine monohydrate supplementation as being advantageous. Previous studies, that have examined the combination of HMB and creatine monohydrate also reported no advantages (or disadvantages) from supplementation when compared to HMB supplementation alone or placebo [33, 34]. Aside from a slight disadvantage for HMB-Cr in reduced peak knee extension torque (fall only), our results agree with those findings. No significant group differences or changes were observed in body composition, circulating concentrations of cortisol and creatine kinase, muscle morphology, knee flexor strength or sprinting performance within either season (i.e., fall season and spring season). Rather, the majority of significant interactions observed in this study were the consequence of differences being detected between seasons (i.e., between fall and spring time points). Specifically, reduced cortisol concentrations (PRE_FALL_ to POST_SPRING_), VL pennation angle (PRE_FALL_ to PRE_SPRING_), and 2nd step force during low-intensity sprinting (fall season to PRE_SPRING_), along with increased knee flexion impulse (PRE_FALL_ to PRE_SPRING_) were noted in athletes who had been consuming HMB-Cr in the fall and were transitioning to Cr. Additionally, several measures of knee flexor strength favored these athletes at PRE_SPRING_. Collectively, the data implies that Division 1-AA rugby players will experience similar results from supplementing with HMB-Cr or Cr during their fall and spring seasons.

Mechanistic research on HMB has demonstrated its influence on promoting muscle protein synthesis and limiting muscle protein breakdown [18–20]. These effects are thought to require a sufficient, damaging stimulus in order to be apparent [30]. In untrained individuals, this requirement is more easily accomplished through exercise and training compared to experienced individuals. Previous research investigating the combined effect of HMB and Cr in elite rugby athletes, participating in concurrent resistance and aerobic training, reported no ergogenic benefits [33, 34]. The authors postulated that the training status of their athletes may have influenced their ability to observe an effect. That is, the protective effects seen with repeated exposure to training may have limited the damage sustained from exercise (i.e., the repeated bout effect) [50], and thus, impaired HMB’s potential for providing a benefit. By comparison, the rugby athletes of the present study were less competitive and theoretically, would have required a lesser stimulus to experience a benefit. However, several factors related to the intensity of the season may have influenced the sufficiency of the stimulus experienced by our athletes. Specifically, the lack of supervised and organized team training resulted in a great deal of individual variability in programming where, though similar between groups, relative intensity could not be determined and suitability was questionable. Supervision by a strength and conditioning coach has been shown to elicit greater adherence and adaptations to sport-specific programming compared unsupervised training [51], and the athletes from the present study did not benefit from the supervision or guidance of such a professional. Moreover, our athletes averaged less than 85% attendance at practice across both seasons and only competed in approximately 70% of available minutes per match. Ultimately, this combination (i.e., strength training and sport-related activities) did not affect cortisol or creatine kinase concentrations during either season. This is noteworthy because rugby athletes are known to experience considerable damage following match play and a season [5], and HMB has been shown to have a curbing effect while also better maintaining strength compared to placebo [21]. In the absence of a sufficient stimulus, there appears to be little value in HMB supplementation. It’s value may have been further diminished by the athletes’ average protein consumption across the season (1.8–4.1 g·kg^− 1^), which was on par with or greater than established recommendations [52]. Regardless, no advantage was seen in comparison to Cr supplementation.

Creatine monohydrate is one of the most widely-used ergogenic supplements for improving lean mass, strength, and sprinting ability [10]. It has been theorized that creatine acts by increasing the availability of energy during exercise [10–12], which would enable greater effort and the opportunity to accumulate more volume to stimulate adaptations. A secondary mechanism responsible for its ergogenic benefits is creatine’s role in hyper-hydrating the skeletal muscle cell [53]. Hyper-hydration of skeletal muscle is known to decrease protein breakdown [15] and increase protein synthesis [16, 17]. In the present study, neither group experienced improvements in lean mass, strength, or sprinting performance within the fall or spring seasons, despite both supplementing with creatine monohydrate. However, improvements in these measures were not expected. Rather, it was thought that the athletes would either maintain or experience decrements due to the demands of the season limiting their ability to train for adaptations. During a rugby season [54] and a soccer pre-season [55], creatine monohydrate supplementation was similar to placebo in that neither supplementation regimen stimulated improvements (or decrements) in body composition or power, respectively. Although the present study was not placebo-controlled, with the exception of HMB-Cr experiencing a reduction in knee extensor strength in the fall, our results agree with those findings. Therefore, based on the assumption that decrements were likely to occur sans supplementation [5] and similar outcomes occurring in each group, it appears that 5 g creatine monohydrate (without HMB) daily was sufficient for the maintenance of body composition and performance in rugby players. Assuming athletes are willing, these findings may be verified in future studies that utilize a true placebo.

The reduction in knee extensor strength observed in HMB-Cr is interesting but likely a spurious observation due to this being the only measure to change within a season. It seems more plausible that this reduction was the consequence of statistical regression, experimental mortality, or a combination of both. Another explanation may be related to caloric and protein intake. Neither relative caloric or protein intake were significantly changed for either group nor did relative protein intake drop below established recommendations [52]. However, both appeared to decrease for HMB-Cr and increase for Cr over the course of the fall season, and nutritional intake between the first- and last-2 weeks of this season is unknown. Nevertheless, any change in nutrition, regardless of significance, that placed an athlete in a deficit for an extended period of time could have impacted force production [52, 56]; though it would seem likely to affect other measures as well. Future investigations might clarify these observations by by accounting for nutritional needs (e.g., measurement of daily metabolic rate, energy expenditure during practice and matches) in addition to the athletes’ intake.

It is noteworthy that the majority of differences observed in this study occurred after the wash-out period and when the cross-over in supplementation was employed. Although differences in dietary and training habits, as well as experimental mortality, may all be responsible here, another explanation for these differences may be related to the effect of the wash-out period itself. Upon discontinuation of creatine monohydrate supplementation, it typically takes a month to eliminate excess concentrations from the body [57] but could be prolonged in individuals who consume a creatine-rich diet [58]. Between the fall and spring seasons, the athletes in the present study were asked to discontinue all supplementation for a 10-week period. The length of this period would have been sufficient to allow intramuscular PCr concentrations to return to baseline, which would affect both energy availability and performance. In contrast, HMB has more commonly been associated with acute supplementation [30] but there is evidence that chronic supplementation (e.g., > 6 weeks) may be required to observe an effect [59]. Since HMB’s ergogenic effect is thought to be myofibrillar in nature (i.e., favorably affecting muscle protein balance) [30], its effects may be more sustainable after ceasing supplementation if nutrition and training remain adequate. Thus, the advantages seen in the athletes transitioning from HMB-Cr (in the fall) to Cr (in the spring) may be indicative of a lingering effect. However, the design of the present investigation can neither support or refute this hypothesis as we did not monitor nutritional intake or training during the wash-out period.

There were several limitations in the present investigation that preclude definitive conclusions from being made at this time. The athletes’ training habits and participation in team-related activities did not result in substantial changes in circulating concentrations of cortisol or creatine kinase, and may have contributed to HMB not presenting an ergogenic benefit. It is possible that concentrations in these markers could have been masked or mitigated by the consumption of nonsteroidal anti-inflammatory drugs [60], but this is speculation. None of the athletes reported regular usage of such drugs during enrollment or while reporting weekly nutritional intake, and midseason and offseason usage was unknown. The lack of damage and effect from HMB may also have been the consequence of creatine monohydrate being sufficient for maintaining lean mass and performance, but this hypothesis could only be supported by the inclusion of a true placebo group. Unfortunately, the athletes in the present study were not willing to discontinue their normal creatine monohydrate supplementation habits for an entire season. Most importantly, the large number of dropouts sustained (i.e., 50% of the initial participant pool completed the entire study) reduced our statistical power to observe group differences. While only one participant reported the supplementation regimen as the reason for leaving the study, the likelihood of losing rugby athletes to injury and leaving the team, particularly at this competitive level, may always be a risk. Future research endeavors may need to recruit a much larger sample to account for this possibility.

## Conclusions

In theory, the combination of HMB with creatine monohydrate should have a greater impact on body composition and performance than either supplement alone due to their having different proposed mechanisms of action [10, 18]. Although existing evidence in rugby players using this combination has not demonstrated an advantage [33, 34], several limitations in their design prevented clear interpretations. This double-blind, cross-over controlled study was designed to account for those limitations but still did not observe an advantage from either supplementation regimen. Lean mass, strength, and sprinting ability were equally maintained following each regimen. Since, the only difference between regimens was the inclusion of 5 g HMB, it appears that Cr supplementation alone will sufficiently maintain body composition and performance in Division 1-AA male, rugby players across their competitive season.

## Data Availability

The datasets used and/or analyzed during the current study are available from the corresponding author on reasonable request.

## Abbreviations

95% C.I.: 95% confidence interval
ATP: Adenosine triphosphate
BF%: Body fat percentage
BMC: Bone mineral content
Cr: 5 g creatine monohydrate + 5 g placebo
ELISA: Enzyme-linked immunosorbent assays
HMB: β-hydroxy β-mehtylbutyrate
HMB-Cr: 5 g HMB + 5 g creatine monohydrate
HPL: Human performance laboratory
ICC: Intraclass correlation coefficients
iDXA: Dual energy x-ray absorptiometry
MVIC: Maximal voluntary isometric contraction
PCr: Phosphocreatine
POST_FALL_: Testing completed following the fall season
POST_SPRING_: Testing completed following the spring season
PRE_FALL_: Testing completed prior to the fall season
PRE_SPRING_: Testing completed prior to the spring season
RF: Rectus femoris
RTD: Rate of torque development
VL: Vastus lateralis

## References

[1] Cunniffe B, Proctor W, Baker JS, Davies B (2009). An evaluation of the physiological demands of elite rugby union using global positioning system tracking software. J Strength Cond Res.

[2] McCann L (2006). Rugby: facts, figures and fun.

[3] Takarada Y (2003). Evaluation of muscle damage after a rugby match with special reference to tackle plays. Br J Sports Med.

[4] Finaud J, Scislowski V, Lac G, Durand D, Vidalin H, Robert A (2006). Antioxidant status and oxidative stress in professional rugby players: evolution throughout a season. Int J Sports Med.

[5] Elloumi M, Ounis OB, Tabka Z, Van Praagh E, Michaux O, Lac G (2008). Psychoendocrine and physical performance responses in male Tunisian rugby players during an international competitive season. Aggress Behav.

[6] Barton BA, Schreck CB, Barton LD (1987). Effects of chronic cortisol administration and daily acute stress on growth, physiological conditions, and stress responses in juvenile rainbow trout. Dis Aquat Org.

[7] Crowley MA, Matt KS (1996). Hormonal regulation of skeletal muscle hypertrophy in rats: the testosterone to cortisol ratio. Eur J Appl Physiol Occup Physiol.

[8] Darmaun D, Matthews DE, Bier DM (1988). Physiological hypercortisolemia increases proteolysis, glutamine, and alanine production. Am J Physiol Endocrinol Metab.

[9] Simmons PS, Miles JM, Gerich J, Haymond MW (1984). Increased proteolysis. An effect of increases in plasma cortisol within the physiologic range. J Clin Invest.

[10] Kreider RB, Kalman DS, Antonio J, Ziegenfuss TN, Wildman R, Collins R (2017). International Society of Sports Nutrition position stand: safety and efficacy of creatine supplementation in exercise, sport, and medicine. J Int Soc Sports Nutr.

[11] Harris R, Hultman E, Nordesjö L-O (1974). Glycogen, glycolytic intermediates and high-energy phosphates determined in biopsy samples of musculus quadriceps femoris of man at rest. Methods and variance of values. Scand J Clin Lab Invest.

[12] Harris R, Edwards R, Hultman E, Nordesjö L, Nylind B, Sahlin K (1976). The time course of phosphorylcreatine resynthesis during recovery of the quadriceps muscle in man. Pflugers Arch.

[13] Essen-Gustavsson B, Tesch P (1990). Glycogen and triglyceride utilization in relation to muscle metabolic characteristics in men performing heavy-resistance exercise. Eur J Appl Physiol Occup Physiol.

[14] Cairns SP (2006). Lactic acid and exercise performance. Sports Med.

[15] Low SY, Rennie MJ, Taylor PM (1996). Modulation of glycogen synthesis in rat skeletal muscle by changes in cell volume. J Physiol.

[16] Berneis K, Ninnis R, Häussinger D, Keller U (1999). Effects of hyper-and hypoosmolality on whole body protein and glucose kinetics in humans. Am J Physiol Endocrinol Metab.

[17] Häussinger D, Gerok W, Roth E, Lang F (1993). Cellular hydration state: an important determinant of protein catabolism in health and disease. Lancet.

[18] Portal S, Eliakim A, Nemet D, Halevy O, Zadik Z (2010). Effect of HMB supplementation on body composition, fitness, hormonal profile and muscle damage indices. J Pediatr Endocrinol Metab.

[19] Wilkinson DJ, Hossain T, Hill D, Phillips B, Crossland H, Williams J (2013). Effects of leucine and its metabolite β-hydroxy-β-methylbutyrate on human skeletal muscle protein metabolism. J Physiol.

[20] Wilkinson DJ, Hossain T, Limb MC, Phillips BE, Lund J, Williams JP (2018). Impact of the calcium form of β-hydroxy-β-methylbutyrate upon human skeletal muscle protein metabolism. Clin Nutr.

[21] Panton LB, Rathmacher JA, Baier S, Nissen S (2000). Nutritional supplementation of the leucine metabolite β-hydroxy-β-methylbutyrate (HMB) during resistance training. Nutrition.

[22] Thomson JS, Watson PE, Rowlands DS (2009). Effects of nine weeks of β-Hydroxy-β-Methylbutyrate supplementation on strength and body composition in resistance trained men. J Strength Cond Res.

[23] Durkalec-Michalski K, Jeszka J, Podgórski T (2017). The effect of a 12-week beta-hydroxy-beta-methylbutyrate (HMB) supplementation on highly-trained combat sports athletes: a randomised, double-blind, placebo-controlled crossover study. Nutrients.

[24] Neighbors K, Ransone J, Jacobson B, Lefavi R (2000). Effects of die-tary β-hydroxy-β-methylbutyrate on body composition incollegiate football players. Med Sci Sports Exerc.

[25] Ransone J, Neighbors K, Lefavi R, Chromiak J (2003). The effect of beta-hydroxy beta-methylbutyrate on muscular strength and body composition in collegiate football players. J Strength Cond Res.

[26] Kreider RB, Ferreira MP, Greenwood M, Wilson M, Grindstaff P, Plisk S (2000). Effects of calcium β-HMB supplementation during training on markers of catabolism, body composition, strength and sprint performance.

[27] Hoffman JR, Cooper J, Wendell M, Im J, Kang J (2004). Effects of beta-hydroxy beta-methylbutyrate on power performance and indices of muscle damage and stress during high-intensity training. J Strength Cond Res.

[28] Kreider R, Ferreira M, Wilson M, Almada A (1999). Effects of calcium â-Hydroxy-â-methylbutyrate (HMB) supplementation during resistance-training on markers of catabolism, body composition and strength. Int J Sports Med.

[29] Slater G, Jenkins D, Logan P, Lee H, Vukovich M, Rathmacher JA (2001). β-Hydroxy-β-methylbutyrate (HMB) supplementation does not affect changes in strength or body composition during resistance training in trained men. Int J Sport Nutr Exerc Metab.

[30] Wilson JM, Fitschen PJ, Campbell B, Wilson GJ, Zanchi N, Taylor L (2013). International Society of Sports Nutrition position stand: beta-hydroxy-beta-methylbutyrate (HMB). J Int Soc Sports Nutr.

[31] Cohen D (1997). The effect of β-hydroxy-β-methylbutyrate (HMB) and resistance training on changes in body composition during positive and negative energy balance-a randomized double-blind study.

[32] Hung W, Liu T-H, Chen C-Y, Chang C-K (2010). Effect of β-hydroxy-β-methylbutyrate supplementation during energy restriction in female judo athletes. J Exerc Sci Fit.

[33] O'Connor D, Crowe M (2003). Effects of [beta]-hydroxy-[beta]-methylbutyrate and creatine monohydrate supplementation on the aerobic and anaerobic capacity of highly trained athletes. J Sports Med Phys Fitness.

[34] O’Connor DM, Crowe MJ (2007). Effects of six weeks of ß-hydroxy-ß-methylbutyrate (HMB) and HMB/creatine supplementation on strength, power and anthropometry of highly trained athletes. J Strength Cond Res.

[35] Wilson GJ, Wilson JM, Manninen AH (2008). Effects of beta-hydroxy-beta-methylbutyrate (HMB) on exercise performance and body composition across varying levels of age, sex, and training experience: a review. Nutr Metab.

[36] McCrory MA, Molé PA, Gomez TD, Dewey KG, Bernauer EM (1998). Body composition by air-displacement plethysmography by using predicted and measured thoracic gas volumes. J Appl Physiol.

[37] Wang Z, Deurenberg P, Guo SS, Pietrobelli A, Wang J, Pierson R (1998). Six-compartment body composition model: inter-method comparisons of total body fat measurement. Int J Obes.

[38] Berg H, Tedner B, Tesch P (1993). Changes in lower limb muscle cross-sectional area and tissue fluid volume after transition from standing to supine. Acta Physiol Scand.

[39] Cadore EL, Izquierdo M, Conceição M, Radaelli R, Pinto RS, Baroni BM (2012). Echo intensity is associated with skeletal muscle power and cardiovascular performance in elderly men. Exp Gerontol.

[40] Chapman DW, Newton M, McGuigan MR, Nosaka K (2008). Comparison between old and young men for responses to fast velocity maximal lengthening contractions of the elbow flexors. Eur J Appl Physiol.

[41] Bemben M (2002). Use of diagnostic ultrasound for assessing muscle size. J Strength Cond Res.

[42] Conchola E, Thompson B, Smith D (2013). Effects of neuromuscular fatigue on the electromechanical delay of the leg extensors and flexors in young and old men. Eur J Appl Physiol.

[43] Thompson BJ, Stock MS, Shields JE, Luera MJ, Munayer IK, Mota JA (2015). Barbell deadlift training increases the rate of torque development and vertical jump performance in novices. J Strength Cond Res.

[44] Aagaard P, Simonsen EB, Andersen JL, Magnusson P, Dyhre-Poulsen P (2002). Increased rate of force development and neural drive of human skeletal muscle following resistance training. J Appl Physiol.

[45] Thompson BJ, Ryan ED, Sobolewski EJ, Conchola EC, Cramer JT (2013). Age related differences in maximal and rapid torque characteristics of the leg extensors and flexors in young, middle-aged and old men. Exp Gerontol.

[46] Mangine GT, McNabb JA, Feito Y, VanDusseldorp TA, Hester GM. Increased resisted sprinting load decreases bilateral asymmetry in sprinting kinetics among rugby players. J Strength Cond Res. 2019. Published ahead of print on February 14, 2020. 10.1519/JSC.0000000000003293 35258269

[47] Bergkvist C, Svensson M, Eriksrud O (2015). Validation of 1080 Sprint and 1080 Quantum. 1080 Motion.

[48] Cohen J (1988). Statistical power analysis for the behavioral sciences.

[49] Rhea MR (2004). Determining the magnitude of treatment effects in strength training research through the use of the effect size. J Strength Cond Res.

[50] McHugh MP (2003). Recent advances in the understanding of the repeated bout effect: the protective effect against muscle damage from a single bout of eccentric exercise. Scand J Med Sci Sports.

[51] Coutts AJ, Murphy AJ, Dascombe BJ (2004). Effect of direct supervision of a strength coach on measures of muscular strength and power in young rugby league players. J Strength Cond Res.

[52] Jäger R, Kerksick CM, Campbell BI, Cribb PJ, Wells SD, Skwiat TM (2017). International society of sports nutrition position stand: protein and exercise. J Int Soc Sports Nutr.

[53] Deminice R, Rosa F, Pfrimer K, Ferrioli E, Jordao A, Freitas E (2016). Creatine supplementation increases total body water in soccer players: a deuterium oxide dilution study. Int J Sports Med.

[54] Chilibeck PD, Magnus C, Anderson M (2007). Effect of in-season creatine supplementation on body composition and performance in rugby union football players. Appl Physiol Nutr Metab.

[55] Claudino JG, Mezêncio B, Amaral S, Zanetti V, Benatti F, Roschel H (2014). Creatine monohydrate supplementation on lower-limb muscle power in Brazilian elite soccer players. J Int Soc Sports Nutr.

[56] Black KE, Black AD, Baker DF (2018). Macronutrient intakes of male rugby union players: a review. Int J Sport Nutr Exerc Metab.

[57] McKenna MJ, Morton J, Selig SE, Snow RJ (1999). Creatine supplementation increases muscle total creatine but not maximal intermittent exercise performance. J Appl Physiol.

[58] Rawson ES, Persky AM, Price TB, Clarkson PM (2004). Effects of repeated creatine supplementation on muscle, plasma, and urine creatine levels. J Strength Cond Res.

[59] Rahimi MH, Mohammadi H, Eshaghi H, Askari G, Miraghajani M (2018). The effects of beta-hydroxy-beta-methylbutyrate supplementation on recovery following exercise-induced muscle damage: a systematic review and meta-analysis. J Am Coll Nutr.

[60] Schoenfeld BJ (2012). The use of nonsteroidal anti-inflammatory drugs for exercise-induced muscle damage. Sports Med.

